# Recognizing and disrupting stigma in implementation of HIV prevention and care: a call to research and action

**DOI:** 10.1002/jia2.25930

**Published:** 2022-07-12

**Authors:** Sarit A. Golub, Rachel A. Fikslin

**Affiliations:** ^1^ Department of Psychology Hunter College New York New York USA; ^2^ Basic and Applied Social Psychology The Graduate Center of the City University of New York New York New York USA; ^3^ Hunter Alliance for Research and Translation Hunter College New York New York USA; ^4^ Einstein‐Rockefeller‐CUNY Center for AIDS Research (ERC‐CFAR) New York New York USA

**Keywords:** stigma, health systems, intersectionality, HIV care continuum, HIV prevention

## Abstract

**Introduction:**

There is robust evidence that stigma negatively impacts both people living with HIV and those who might benefit from HIV prevention interventions. Within healthcare settings, research on HIV stigma has focused on intra‐personal processes (i.e. knowledge or internalization of community‐level stigma that might limit clients’ engagement in care) or inter‐personal processes (i.e. stigmatized interactions with service providers). Intersectional approaches to stigma call us to examine the ways that intersecting systems of power and oppression produce stigma not only at the individual and interpersonal levels, but also within healthcare service delivery systems. This commentary argues for the importance of analysing and disrupting the way in which stigma may be (intentionally or unintentionally) enacted and sustained within HIV service implementation, that is the policies, protocols and strategies used to deliver HIV prevention and care. We contend that as HIV researchers and practitioners, we have failed to fully specify or examine the mechanisms through which HIV service implementation itself may reinforce stigma and perpetuate inequity.

**Discussion:**

We apply Link and Phelan's five stigma components (labelling, stereotyping, separation, status loss and discrimination) as a framework for analysing the way in which stigma manifests in existing service implementation and for evaluating new HIV implementation strategies. We present three examples of common HIV service implementation strategies and consider their potential to activate stigma components, with particular attention to how our understanding of these dynamics can be enhanced and expanded by the application of intersectional perspectives. We then provide a set of sample questions that can be used to develop and test novel implementation strategies designed to mitigate against HIV‐specific and intersectional stigma.

**Conclusions:**

This commentary is a theory‐informed call to action for the assessment of existing HIV service implementation, for the development of new stigma‐reducing implementation strategies and for the explicit inclusion of stigma reduction as a core outcome in implementation research and evaluation. We argue that these strategies have the potential to make critical contributions to our ability to address many system‐level form stigmas that undermine health and wellbeing for people living with HIV and those in need of HIV prevention services.

## 1 INTRODUCTION

HIV stigma negatively impacts both people living with HIV and those who might benefit from HIV prevention interventions [1, 2, 3, 4]. The vast majority of research on HIV stigma in healthcare has focused on *intra‐personal processes* (i.e. the ways in which the internalization of community‐level stigma affects clients’ engagement in care) or *inter‐personal processes* (i.e. stigma in provider–client interactions) [4, 5, 6, 7]. Limited research has examined the extent to which intra‐personal and inter‐personal processes are exacerbated by programmatic or systemic factors, including the way in which HIV prevention and care are delivered.

Public health researchers are increasingly recognizing the importance of intersectionality as a framework for understanding the ways in which healthcare systems create and sustain health inequities [8, 9, 10, 11]. Intersectional approaches call us to examine not only individuals’ experience of stigma at the intersections of systems of power and oppression, but also the policies, processes and protocols that create stigmatizing environments for clients [12, 13]. A central premise of stigma theory is that stigma occurs in situations in which power is exercised [14]. The implementation of healthcare involves an inherent power imbalance between client and provider/system because the client is entirely subject to rules about how, when, where and to whom care is provided or denied. These power dynamics intersect with social systems of power and oppression, such that those with power and control within healthcare systems are disproportionately privileged along lines of race, class and education [15], whereas clients in need of HIV services are disproportionately marginalized by those systems [16]. Intersectional and multi‐level frameworks argue that health inequity is perpetuated through interactions between multiple sites and levels of power [12, 17] and that this power is exercised in the context of social institutions [8, 17, 18, 19]. HIV service implementation is one domain in which power is exercised in ways that perpetuate stigma at the intersection of social hierarchies.

This commentary argues for the importance of analysing and disrupting the way in which stigma may be enacted and sustained within HIV service implementation, by which we mean the policies, protocols and strategies used to deliver HIV prevention and care. HIV service implementation includes strategies that affect care delivery, including policies that determine how healthcare is organized, protocols that govern aspects of care delivery, such as treatment, testing and education, and procedures that define client–provider interactions. We contend that as HIV researchers and practitioners, we have failed to fully specify or examine the mechanisms through which HIV service implementation *itself* may reinforce stigma. This gap is a major limitation in our ability to address and rectify many system‐level stigmas that undermine health and wellbeing for people living with HIV and those with prevention needs.

Link and Phelan's operationalization of five stigma components [14] provides a useful framework for analysing the way in which stigma may manifest in existing HIV service implementation and for evaluating new strategies as stigmatizing or stigma reducing. Below, we define these components and describe how they help identify the sources of intersectional stigma using three examples of HIV service implementation strategies—the use of risk‐based algorithms to determine eligibility [20], the segregation of HIV services from other health services [21, 22] and the adoption of protocols that present logistical hurdles to receiving care [23]. We then provide guiding questions (Table 1) for use in the evaluation of new and existing HIV service implementation strategies in the context of intersectional stigma.

**Table 1:** Questions for assessing HIV service implementation strategies for stigma

Stigma components	Examples of HIV service implementation strategies that might activate stigma components^a^	Questions for analysing and assessing existing HIV service implementation strategies	Questions for elevating an intersectional approach to combatting HIV service implementation stigma	Questions for developing and testing new HIV service implementation strategies
Labelling (i.e. classifying a particular condition or attribute as “different,” and assigning a specific marker to communicate that difference in society)	Eligibility screens or protocols that label certain behaviours or people as “high‐risk” Protocols that seek to identify certain clients as at risk for non‐adherence Visual disclosure of client's HIV or medication status on charts or other paperwork Protocols that rely on provider discretion to offer HIV testing or other services Outreach efforts that “target” certain individuals or communities	Does this strategy label certain behaviours or groups as relevant for HIV prevention and care and leave others out? Does it label those with HIV or those who need HIV prevention?	Do labelling practices within HIV prevention and care label certain groups more than others with respect to race, gender, sexuality, class, ability, immigration status and/or other intersecting dimensions of power and oppression?	Does this strategy decrease the need or opportunity to label clients in ways that may activate or reinforce stereotypes?
Stereotyping (i.e. linking labelled conditions or attributes to negative or undesirable characteristics)		Does this strategy reinforce stereotypes about who gets HIV or who is at risk for HIV? Does it rely on stereotypes about which types of clients are more/less likely to adhere to treatment or return for visits?	Does this programme, practice or policy reinforce stereotypes about people living with HIV or clients at risk for HIV at intersections of race, gender, sexuality, class, ability, immigration status and/or other intersecting dimensions of power and oppression?	Does this strategy reduce provider reliance on stereotypes to identify clients or make decisions about their care?
Separation (i.e. physical, linguistic or other segregation of labelled individuals from the rest of society)	Segregation of clinics, days/times, entrances or procedures for HIV treatment or prevention services Protocols that offer HIV‐related services only to certain clients Failure to integrate HIV services into existing care (e.g. primary care or OBGYN) Creation of separate programmes for HIV prevention and care needs related to sexual health versus substance use	Does this strategy separate HIV services from other types of care? Does it separate people living with HIV or those with HIV prevention needs from other clients?	Do these separations create or reinforce segregation on the basis of race, gender, sexuality, class, ability, immigration status and/or other intersecting dimensions of power and oppression?	Does this strategy allow for greater integration of HIV services into mainstream or “normalized” care provision? If HIV‐related services remain segregated, does the value of this segregation outweigh the potential cost? Have efforts been made to ensure that this separation is as de‐stigmatizing as possible?
Status loss (i.e. devalued placement in a social hierarchy that confers disadvantage)	Absence of policies against abuse or harassment of people living with HIV or clients seeking prevention Absence of policies that protect clients from disclosure of health information Absence of policies that promote gender‐affirmation (e.g. name/pronoun checks and gender‐neutral bathrooms) Protocols that place logistical or financial burdens on patients to receive prescriptions Appointment times and visit structures that favour clients with flexible schedules and time/money for multiple visits Failure to provide adequate translation services	Does this strategy place an undue burden on people living with HIV or other HIV service clients compared to others? Does it dehumanize or otherwise devalue certain clients?	Does this strategy reinforce existing patterns of inequity on the basis of race, gender, sexuality, class, ability, immigration status and/or other intersecting dimensions of power and oppression?	Does this strategy rectify or address existing barriers to care that have historically marginalized certain clients or populations?
Discrimination (i.e. explicit or implicit devaluation, rejection, exclusion or mistreatment)		Does this strategy favour certain groups of clients over others?	Even if applied equally to all clients, are the outcomes of this strategy equitable on the basis of race, gender, sexuality, class, ability, immigration status and/or other intersecting dimensions of power and oppression?	Does this strategy proactively sanction discriminatory behaviour among providers and incentivize supporting and valuing clients?

^a^Examples may apply to multiple domains of stigma simultaneously.

## 2 DISCUSSION

Link and Phelan define stigma as the convergence of five inter‐related components: labelling, stereotyping, separation, status loss and discrimination [14]. *Labelling* refers to the recognition of a particular condition or attribute as “different” and the assignment of a specific marker to communicate that difference in society. *Stereotyping* refers to a process in which these labelled differences are linked to negative or undesirable characteristics. Labelling and stereotyping operate together, but the recognition of labelling as a discrete stigma process underscores the fact that stigma results from the social construction of categories, rather than inherently valid distinctions. *Separation* refers to the process through which social labels and their stereotypes lead to a separation between “those people” and the rest of society. *Status loss* and *discrimination* refer to the ways in which labelling, stereotyping and separation lead to explicit actions that exclude and mistreat stigmatized groups. Status loss refers specifically to individuals’ devalued placement in a social hierarchy, which often results in lower status individuals needing to expend additional effort and resources than higher status individuals to have their needs met. Status loss is a source of discrimination, but discrimination extends to other behaviours at the interpersonal, organizational or structural levels that disadvantage stigmatized populations.

In Table 1, we provide examples of how each component may manifest in HIV service implementation, along with specific questions corresponding to each component that can be used to assess the extent to which HIV service implementation strategies inadvertently activate stigma. For example, one common practice in HIV service provision is the use of “high‐risk” screening algorithms to determine which clients are offered HIV testing, pre‐exposure prophylaxis (PrEP) or other services [20, 24, 25]. This process places a negative *label* on specific behaviours (e.g. age of sexual debut, number of sexual partners, condomless anal sex and substance use) that may be fundamental to clients’ identity, relationships or personal fulfilment. Individuals screened using these algorithms may feel that their behaviour is being judged, shamed or pathologized [26]. The concept of “high‐risk” behaviours, individuals or populations evokes powerful *stereotypes*, which have consistently fuelled prejudice and discrimination within healthcare settings [27, 28]. As we (SAG) have written previously, risk‐focused algorithms reinforce stereotypes and negative client perceptions among providers, which contribute to reluctance to offer prevention interventions to clients in need [29, 30, 31].

In column 4 of Table 1, we provide a series of questions for each stigma component to guide reflection on how HIV service implementation strategies may activate intersectional stigma and affect clients’ care in different ways based on their social positioning within intersecting systems of power and oppression [32, 33]. An intersectional approach to HIV stigma begins by examining its interaction with other forms of societal stigma, for example sexism, heterosexism, racism and classism [12, 33, 34], and the ways these systems determine who is most vulnerable to and negatively impacted by HIV [16, 35, 36] and who is most able to benefit from existing HIV service implementation [37]. Additionally, HIV is frequently experienced in the context of other stigmatized health conditions and behaviours, such as substance use and sexual behaviour, which are themselves situated in intersecting power systems [12, 33].

Returning to our example of risk‐based algorithms for determining HIV service eligibility, an intersectional lens helps us analyse why and for whom this practice might be stigmatizing. In the United States, negative sexual stereotypes about sexual minority men intersect with negative sexual stereotypes for Black and Latinx individuals in the context of heterosexist and racial marginalization [38, 39, 40]. Thus, sexual minority men of colour may be more likely to experience risk‐focused assessments as stereotyping, contributing to harmful healthcare experiences. On the other hand, behaviour‐based risk screens may fail to identify cisgender heterosexual women as in need of HIV‐related services, because they neglect structural, community and network factors that affect HIV acquisition [36, 41]. Because Black women comprise a disproportional percentage of new HIV diagnoses in the United States [16, 42], practices that neglect women perpetuate disparities at the intersection of racism, sexism and homophobia. Screening practices that emphasize labelling and stereotyping of “high‐risk” individuals can contribute to discrimination and status loss for individuals with negatively stereotyped identity intersections and neglected identity intersections.

The final column of Table 1 provides questions for evaluating the extent to which new strategies mitigate against stigma to reduce health inequities. For example, as we develop strategies for increasing intervention uptake, it is important to consider the extent to which promulgation of stereotypes related to who “needs” HIV prevention and care may motivate individuals to underestimate their own need for these services to distance themselves from such stereotypes. Clients’ risk perception is often unrelated to provider assessment of “objective” risk using screening tools, but is strongly negatively associated with perceived stigma [43, 44]. The stigmatizing nature of this method of screening may discourage clients from disclosing their relevant behaviours and identities to avoid being labelled or stereotyped and potentially discriminated against. While this has not specifically been tested, there is evidence that stigma can affect identity disclosure, which can impact HIV service provision [45]. Independent of behavioural eligibility, HIV stigma has been negatively associated with both testing behaviour and willingness to consider PrEP [27, 46, 47]. Questioning the extent to which an implementation strategy does or does not label clients or increase stereotyping may help create new stigma‐mitigating strategies and promote increased access, uptake and sustainment of HIV services.

Another common HIV service implementation practice that may be unintentionally perpetuating stigma is the separation of HIV services from other service provision, including primary care, Obstetrics/Gynecology care or even sexually transmitted infection testing and treatment [21, 22]. There are several rationales for developing HIV‐specific care programmes—protection of people living with HIV from HIV stigma in mainstream care settings, increasing community among clients living with HIV or ensuring that all providers in a care setting are experts in HIV care. However, the definition of separation as a core component of stigma requires us to consider the potential stigmatizing impacts of this implementation strategy. Applying Link and Phelan's framework [14], the continued *separation* of HIV services from other forms of healthcare *labels* HIV as fundamentally “different” from other healthcare needs and reinforces *stereotypes* that those in need of HIV‐related services are qualitatively distinct from other clients. It also has the potential to confer *status loss*, by requiring people living with HIV or those needing HIV prevention services to expend additional time, effort and resources to access both these services and other needed healthcare services.

Applying an intersectional lens, this separation fails to acknowledge clients’ complex health experiences and the interaction between HIV and other medical conditions that disproportionately impact those who are most marginalized in a particular socio‐political context [12, 48, 49]. The burden of seeking separated care may be especially harmful considering that those with less access to HIV prevention services and HIV education, such as those in rural communities and burdened by class oppression, are disproportionately likely to have comorbid healthcare needs and more adverse HIV outcomes [50, 51, 52].

Taking HIV stigma mitigation seriously in the development of new HIV service implementation strategies requires us to reconsider the utility of limiting HIV services to separate healthcare sites, certain times/days or specialized personnel. Several studies conducted in sub‐Saharan Africa have demonstrated that the integration of HIV care into primary healthcare, sometimes called decentralization, can improve clients’ satisfaction with HIV education, increase willingness to accept HIV services, increase HIV care enrolment and increase client HIV care sustainment over time [53, 54, 55, 56, 57]. Comparatively, decentralization of HIV service provision may enable clients to navigate care without being labelled by people in their community as having HIV, which could lessen the experiences of discrimination and give people more control over disclosure [58].Therefore, when stigma in the forms of labelling, segregation and discrimination is reduced at the HIV services implementation level, it may in turn reduce labelling, segregation and discrimination at the interpersonal and community level for people living with HIV.

A third example of applying the five stigma components to analyse stigma in HIV service implementation is a consideration of the logistical barriers that clients must navigate in order to access care. While systems‐level barriers are recognized as a critical issue, limited resources are devoted to changing clinic hours to increase accessibility, providing care in multiple languages or hiring client navigators who might help with transportation, childcare or other needs. Such systemic issues are often not considered explicitly stigma‐related barriers to care, which is a missed opportunity for acknowledging the ways in which logistical barriers confer *status loss* for clients in need of care [59].

Relatedly, there had been increasing attention to the need for “immediate start” of anti‐retroviral treatment or PrEP, in order to better support people recently diagnosed with HIV [59, 60, 61, 62, 63]. But in most settings, receipt of a prescription for HIV treatment or PrEP requires clients to attend multiple clinical visits, some of which are explicitly designed to assess whether they are likely to return for more clinical visits in the future [64]. Once clients are prescribed medication, refills may be restricted if they fail to return for testing and clinical visits at specific intervals [23, 65]. Additionally, there is often an emphasis on identifying clients who are likely to miss clinic visits and to consider placing additional restrictions on their access to medications [66]. Clients who are already marginalized on the basis of race, class or other experiences, such as substance use, are most likely to be labelled, stereotyped, denied services or blamed for their “failure” to sustain care, which can reinforce negative racial and class‐based stereotypes [67, 68, 69].

Reframing implementation strategies that reduce logistical burdens and gatekeeping as stigma‐reduction interventions may be particularly motivating for research and practice. For example, there is widespread recognition that frequent appointment requirements for HIV care and quarterly refill requirements for PrEP are extremely burdensome for clients [59, 70, 71, 72], but there has been little empirical assessment of whether allowing longer intervals between appointments and prescription refills would have any impacts on safety, efficacy or clinical outcomes. Reconsidering clinical protocols through the lens of stigma reducing, client‐centred care may focus attention on innovative strategies that reduce medical gate‐keeping and communicate to clients that facilitating their access to HIV prevention and care is valued. Importantly, the pathway to designing affirming, inclusive and stigma‐reducing care necessitates listening to and centring the voices of those most affected by intersectional HIV stigma and committing to transforming the healthcare systems we have now to the healthcare systems marginalized people need. Further, these processes for evaluating and developing destigmatizing healthcare services are not only relevant for HIV prevention and care, but for all types of healthcare services, especially those designed for stigmatized health conditions.

## 3 CONCLUSIONS

This commentary is intended to be a theory‐informed call to action for the assessment of HIV service implementation, for the development of new stigma‐reducing implementation strategies and for the explicit inclusion of stigma reduction as a core outcome in implementation research. We encourage researchers and practitioners to consider the insidious (and often unintentional) activation of stigma components in specific protocols, policies, programmes and service organization. We also encourage the application of intersectionality as a theoretical and methodological framework for greater understanding of the impact of HIV services implementation on the lives of people living with HIV and those in need of prevention in the context of intersecting systems of power and oppression. Using the questions in Table 1, we can begin to identify the ways that services implementation perpetuates stigma for those disproportionately burdened by the HIV epidemic and develop new strategies that transform healthcare systems in service of health equity.

## FUNDING

This work was supported, in part, by the Einstein‐Rockefeller‐CUNY Center for AIDS Research (P30‐AI124414; Goldstein, PI). R01MH123262; funded by NIMH, with Sarit Golub and Kathrine Meyers, MPI.

## COMPETING INTERESTS

The authors declare no competing interests.

## AUTHORS’ CONTRIBUTIONS

Both SAG and RAF developed the concept for this paper and contributed to writing, revision and figure development.
